# Subcutaneous nitroglycerin increased the success rate of radial artery cannulation in women with gestational hypertension undergoing cesarean section: A randomized controlled trial

**DOI:** 10.1007/s00101-023-01264-6

**Published:** 2023-03-08

**Authors:** Xin Men, Qian Wang, Pei Chen, Wen-sheng Hu, Yun Chai, Hong-yan Shou, Zhen-feng Zhou

**Affiliations:** 1 Department of Anesthesiology, Hangzhou Women’s Hospital (Hangzhou Maternity and Child Health Care Hospital, Hangzhou First People’s Hospital Qianjiang New City Campus, The Affiliated Women’s Hospital of Hangzhou Normal University), 315014 Hangzhou, China; 2 Department of Anesthesiology, The Affiliated ZheJiang Hospital, School of Medicine, Zhejiang University, 315014 Hangzhou, China

**Keywords:** Nitroglycerin, Radial artery puncture, Pregnancy, Hypertension, Ultrasound-guided, Nitroglyzerin, Radialarterienpunktion, Schwangerschaft, Hypertonie, Ultraschallgesteuert

## Abstract

**Background:**

Radial artery cannulation helps to maintain the stability of maternal hemodynamics and reduce complications; however, it is difficult for women with gestational hypertension. Subcutaneous nitroglycerin was found to improve the first attempt success rate of radial artery cannulation in pediatric patients. Therefore, this study evaluated the effect of subcutaneous nitroglycerin on the radial artery diameter and area, blood flow rate and the success rate of radial artery cannulation in women with pregnancy-induced hypertension.

**Methods:**

A total of 94 women with gestational hypertension and risk of intraoperative bleeding undergoing cesarean section were identified and randomized into the subcutaneous nitroglycerin group and control group. The primary outcome was the success rate of left radial artery cannulation within 3 min after subcutaneous injecting (T2). The puncture time, number of attempts, the overall complications, and ultrasonographic measurements including radial artery diameter, cross-sectional area and depth were also recorded before subcutaneous injection (T1), 3 min after subcutaneous injection (T2) and immediately after radial artery cannulation (T3).

**Results:**

The first attempt success rate of radial artery cannulation was significantly higher (97.9% vs. 76.6%, *p* = 0.004) and procedure time to success was significantly shorter (111 ± 18 s vs. 171 ± 70 s, *p* < 0.001) in the subcutaneous nitroglycerin group as compared to the control group. The subcutaneous nitroglycerin group also had a significantly less overall number of attempts as 1/2/3 attempts (*n*), 46/1/0 vs. 36/7/4 (*p* = 0.008). Compared with the control group, the diameter and cross-sectional area of radial artery increased significantly at the T2 and T3 points in the subcutaneous nitroglycerin group (*p* < 0.001), as well as percentage change of radial artery diameter and CSA. Vasospasm (6.4% vs. 31.9%; *p* = 0.003) was significantly lower in the subcutaneous nitroglycerin group; however, no difference was found in hematoma (2.1% vs. 12.8%; *p* = 0.111).

**Conclusion:**

Subcutaneous nitroglycerin along with the routine local anesthetic preparation before radial artery cannulation increased the first attempt success rate of radial artery cannulation and decreased the overall number of cannulation attempts in women with gestational hypertension and risks of intraoperative bleeding undergoing cesarean section, it also decreased cannulation times and overall number of vasospasms.

**Supplementary Information:**

The online version of this article (10.1007/s00101-023-01264-6) contains one additional figure.

## Abstract (de)

**Hintergrund:**

Die Kanülierung der A. radialis trägt zur Aufrechterhaltung der maternalen Hämodynamik und zur Verringerung von Komplikationen bei. Bei Frauen mit Schwangerschaftshypertonie ist dies jedoch schwierig. Es wurde festgestellt, dass subkutanes Nitroglyzerin die Erfolgsquote beim ersten Versuch der Kanülierung der A. radialis bei pädiatrischen Patientinnen verbessert. Daher wurde in dieser Studie die Wirkung von subkutanem Nitroglyzerin auf den Durchmesser und die Fläche der A. radialis, die Blutflussrate und die Erfolgsrate der Kanülierung der A. radialis bei Frauen mit schwangerschaftsbedingter Hypertonie untersucht.

**Methoden:**

Insgesamt 94 Frauen mit Schwangerschaftshypertonie und dem Risiko einer intraoperativen Blutung, die sich einem Kaiserschnitt unterziehen mussten, wurden identifiziert und nach dem Zufallsprinzip in eine Gruppe mit subkutanem Nitroglyzerin und eine Kontrollgruppe eingeteilt. Als primäres Ergebnis wurde die Erfolgsrate der Kanülierung der linken Radialarterie innerhalb von 3 min nach der subkutanen Injektion (T2) ermittelt. Die Punktionszeit, die Anzahl der Versuche, die Gesamtkomplikationen und die sonographischen Messungen einschließlich des Durchmessers der Radialarterie, der Querschnittsfläche und der Tiefe wurden ebenfalls vor der subkutanen Injektion (T1), 3 min nach der subkutanen Injektion (T2) und unmittelbar nach der Kanülierung der Radialarterie (T3) aufgezeichnet.

**Ergebnisse:**

Die Erfolgsquote beim ersten Versuch der Kanülierung der A. radialis war in der subkutanen Nitroglyzerin-Gruppe im Vergleich zur Kontrollgruppe signifikant höher (97,9 % vs. 76,6 %; *p* = 0,004) und die Zeit bis zum Erfolg war signifikant kürzer (111 ± 18 s vs. 171 ± 70 s; *p* < 0,001). In der subkutanen Nitroglyzerin-Gruppe war auch die Gesamtzahl der Versuche signifikant geringer (1/2/3 Versuche [*n*], 46/1/0 vs. 36/7/4; *p* = 0,008). Im Vergleich zur Kontrollgruppe nahmen der Durchmesser und die Querschnittsfläche der A. radialis an den Punkten T2 und T3 in der subkutanen Nitroglyzerin-Gruppe signifikant zu (*p* < 0,001), ebenso wie die prozentuale Veränderung des Durchmessers und des CSA der A. radialis. Der Vasospasmus (6,4 % vs. 31,9 %; *p* = 0,003) war in der subkutanen Nitroglyzerin-Gruppe signifikant geringer. Es wurde jedoch kein Unterschied in Bezug auf Hämatome festgestellt (2,1 % vs. 12,8 %; *p* = 0,111).

**Schlussfolgerung:**

Subkutanes Nitroglyzerin zusammen mit der routinemäßigen Vorbereitung auf die Lokalanästhesie vor der Kanülierung der A. radialis erhöhte die Erfolgsrate beim ersten Versuch der Kanülierung der A. radialis und verringerte die Gesamtzahl der Kanülierungsversuche bei Frauen mit Schwangerschaftshypertonie und dem Risiko einer intraoperativen Blutung, die sich einem Kaiserschnitt unterziehen. Außerdem verringerte es die Kanülierungszeiten und die Gesamtzahl der Gefäßspasmen.

## Introduction

Globally, about 10% of women have pregnancy-induced hypertension during pregnancy, and hypertensive disease is still a risk factor for maternal and neonatal morbidity and mortality [1, 2]. The incidence of pregnancy-induced hypertension may increase as the increased incidence of obesity, maternal age and comorbidities [1]. Women with gestational hypertension should receive the same standards of critical care as other acutely ill patients [1]. Radial artery cannulation was a common operation in critical care, which helps to maintain the stability of maternal hemodynamics and reduce complications. Radial artery cannulation has several advantages, including easy compressibility, superficial location, distance from vital nerves, and low rate of procedural complications [3, 4].

However, it is difficult for women with pregnancy-induced hypertension as the basic pathological feature of pregnancy-induced hypertension is systemic arteriole spasm [1]. Although ultrasound guidance can significantly increase radial artery cannulation success rate [5–7], the success rate of initial cannulation is still varied from 51% to 95%. Furthermore, the success rate of initial cannulation in patients with gestational hypertension is not clear [8–12].

Nitroglycerin is an effective vasodilator and topical nitroglycerin has been used to dilate arteries [13, 14]. A recent study even found that subcutaneous nitroglycerin improved the radial artery cannulation success rate in pediatric patients [15]; however, there was no evidence for women with gestational hypertension. Therefore, this study aimed to evaluate the effectiveness and safety of subcutaneous nitroglycerin on radial artery puncture in women with gestational hypertension.

## Methods

### Study design

The Ethics Committee of Hangzhou Women’s Hospital approved this study (IRB:2021-K(9)-02). This prospective, randomized, and double-blind trial was conducted from 3 January 2022 to 6 June 2022 after received written informed consent from all participants and finally 94 women were recruited.

Women with gestational hypertension and risks of intraoperative bleeding should receive intraoperative invasive arterial blood pressure monitoring undergoing elective cesarean section will be selected. Gestational hypertension includes mild preeclampsia and severe preeclampsia. Severe preeclampsia was defined as a blood pressure of 160/100 mm Hg or greater on two occasions at least 4 h apart while the patient was on bed rest. Risks of intraoperative bleeding were placental abruption, placental implantation, multiple cesarean sections according to the obstetrican’s judgment.

The exclusion criteria were emergency surgery, negative modified Allen’s test, infection or external injury at or near the puncture site, coagulopathy, and vascular diseases such as vasculitis.

Standard monitoring was applied, and we suggested applying intraoperative invasive arterial blood pressure monitoring for women with gestational hypertension and risks of intraoperative bleeding during cesarean section at our medical institution. All women were supine with the arm extended to 60° and supported on the arm plate during artery cannulation and ultrasonographic measurements.

### Randomization and blinding

The pregnant women were randomly divided into a control group and a nitroglycerin group at a 1:1 ratio by an independent researcher using numbered sealed envelopes. An independent researcher performed nitroglycerin injection or local anesthesia and was not involved in data collection. A doctor with much experience in vascular examinations performed the ultrasound examinations and was blind to the group allocation. The outcome assessor who was unaware of the group allocation evaluated the cross-sectional area and diameter of the radial artery according to ultrasonographic measurements. In addition, the included patients, surgical doctors, and data analysts were all blinded to the group assignments.

### Subcutaneous nitroglycerin preparation and injection

A solution of lidocaine 0.2 ml of 0.8% lidocaine combined with 300 µg (0.3 ml) nitroglycerin (1 mg/ml) was subcutaneously injected in the nitroglycerin group, and 0.5 ml of 0.8% lidocaine without nitroglycerin was subcutaneously injected in the control group. Dosing was slowly administered for more than 5 s to avoid systemic vasodilation.

### Ultrasonographic measurements

Baseline measurements (T1) were performed before subcutaneous injection. All variables were measured again at 3 min after subcutaneous injection (T2) and immediately after radial artery cannulation (T3).

**Fig. 1:**
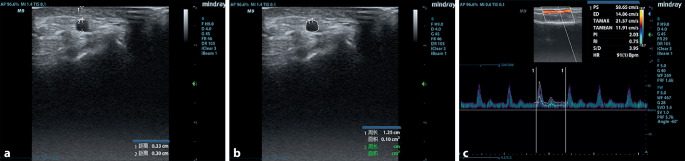
Ultrasonographic measurements: **a** internal diameter and depth (cm), **b** cross-sectional area (cm^2^, CSA), **c** the time average maximal velocity (TAMAX, cm/s)

The ultrasonic probe was placed longitudinally over the radial artery, and ultrasonographic measurements were recorded close to 2 cm from the radial styloid process. A mindray machine (M9, Shenzhen, Guangdong, China) with a 4–12 MHz variable frequency linear array transducer and a 1–5 MHz variable frequency convex array ultrasound transducer were applied. A set of vital signs and ultrasonographic parameters, such as internal diameter (cm, Fig. 1a), cross-sectional area (cm^2^, CSA) of radial artery (Fig. 1b) and depth (cm, Fig. 1a) under short axis (out of plane) view, were measured [5, 16]. Radial artery diameter is measured as the vertical distance between the inner walls of an artery by an electronic caliper on a machine [17], and the image corresponding to the end diastole is located by freezing the image at the end diastole. Radial artery depth is measured as the distance between the transducer and the near edge of the artery.

After activating pulsed wave Doppler ultrasound (PWD) mode, the volumetric gate was placed in the center of the arterial lumen, the arterial hemodynamics parameters were recorded. The ultrasound angle was kept between 30° and 60° in the process of the examination, which was defined as the angle between the direction of blood flow and the ultrasound beam. The time average maximal velocity (TAMAX, cm/s) was recorded as shown in Fig. 1c. According to a previous study [18], the volume of blood flow (ml/s) was also calculated as CSA × TAMAX (Fig. 1c).

### Ultrasound-guided radial artery cannulation

The left radial artery was chosen and the puncture point was close to 2 cm from the radial styloid process. The operator performed ultrasound-guided puncture at 3 min after the intervention/control injection and the puncture time was recorded.

We applied the out-of-plane without guide wire technique and a 20-gauge arterial catheter to perform arterial cannulation [19, 20]. During ultrasound-guided arterial catheterization, the operator was not allowed to intentionally puncture either wall of the artery. However, guide wires were not allowed in order to eliminate the effect of other factors on outcomes. It was assumed that the cannulation had been completed when the arterial waveform was shown on the monitor. It was considered to have failed if cannulation was not accomplished in 10 min [15]. After the failure, a contralateral radial artery was used for cannulation without subcutaneous injection. The total operation time of arterial cannulation was recorded as the time between the first puncture of the skin with the needle catheter and the arterial waveform was shown on the monitor. We also recorded intraoperative complications, including hematoma and vasospasm, which were defined as the radial artery diameter decreasing by more than 25% after cannulation [4, 21–26]. The patients’ basic documentation was collected, including age, body mass index, and the American Society of Anesthesiologists physical (ASA) condition classification.

### Primary outcome

The primary outcome was defined as the first attempt success rate of radial artery cannulation within 3 min after injecting. Secondary results included the puncture time, number of attempts, radial artery diameter, cross-sectional area and depth at T1, T2 and T3 points, and the overall complications.

### Sample size

The radial artery cannulation success rate was 91.2% and 66.1% in the subcutaneous nitroglycerin group and the control group, respectively [15]. A two-tailed χ2 test with a type I error probability of 0.05 was performed and 94 patients were estimated to provide 80% power (G-Power version 3.1; The Institute for Experimental Psychology in Dusseldorf, Germany).

### Statistical analysis

Categorical variables were expressed as frequency (percentage age), normally and nonnormally distributed variables were expressed as the mean ± SD and the medians with interquartile ranges, respectively. The Pearson test was used for the correlation analysis. Between the groups and the different times, repeated measurements data were analyzed by repeated measured variables. Differences between the groups measured at the same time points were performed using Student’s two-sample t test or the Mann-Whitney U test. The limit of statistical significance was set to *p* < 0.05 when two-sided *p* values are performed (SPSS V. 18.0, XXX, Chicago, IL, USA).

## Results

### Baseline clinical parameters

**Fig. 2:**
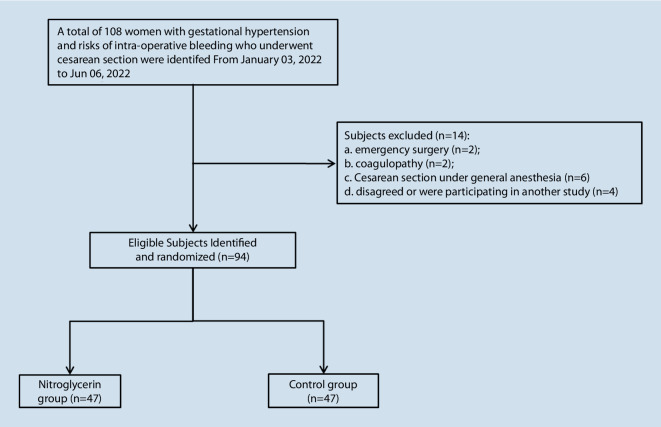
Study flowchart of population recruitment

Of 108 women with gestational hypertension and risks of intraoperative bleeding who underwent cesarean section, 94 patients were identified and completed the follow-up, and 14 patients met the exclusion criteria as shown in Fig. 2. The basic parameters of the two groups are summarized in Table 1, and no difference was found.

**Table 1:** Patient characteristics of control group and nitroglycerin group for radial artery cannulation

	Control (*N* = 47)	Nitroglycerin (*N* = 47)	*p*-value
Age (years)	30 ± 4	31 ± 3	0.700
Estimated gestational age (weeks)	37.3 ± 1.3	37.6 ± 1.3	0.248
Body mass index (kg/m^2^)	28 ± 4	27 ± 3	0.163
Baseline systolic pressure (mm Hg)	134 ± 15	134 ± 13	0.934
Baseline diastolic pressure (mm Hg)	78 ± 12	77 ± 10	0.797
Baseline heart rate (bpm)	85 ± 12	80 ± 16	0.079
Gestational hypertension	–	–	0.759
Mild preeclampsia	40 (85.1%)	42 (89.4%)	–
Severe preeclampsia	7 (14.9%)	5 (10.6%)	–
Antihypertension treatment	–	–	0.775
Labetolol	17 (36.2%)	14 (29.8%)	–
Nicardipine	7 (14.9%)	7 (14.9%)	–
Nifedipine	0 (0%)	0 (0%)	–
Magnesium	4 (8.5%)	7 (14.9%)	–

Values are mean ± SD or number (proportion)

The average age of the study population was 31 years, the average body mass index (BMI) was 28, the average estimated gestational age was 38 weeks, 12.8% had severe preeclampsia, and the other patients had mild preeclampsia.

Over time, no statistically significant differences were observed in blood pressure and HR (heart rate) between the two groups (Supplemental Fig. 1).

### Radial artery cannulation

Compared to the control group, the first attempt success rate of radial artery cannulation was significantly higher (97.9% vs. 76.6%, *p* = 0.004) and procedure time to success within the first attempt was significantly shorter (111 ± 18 s vs. 171 ± 70 s, *p* < 0.001) in the nitroglycerin group. The nitroglycerin group also had a significantly lower overall number of attempts (1/2/3 attempts *n*, 46/1/0 vs. 36/7/4, *p* = 0.008). However, there was no difference in second attempt success rate within 10 min (100% vs. 91.5%; *p* = 0.117) between the two groups. Vasospasm (6.4% vs. 31.9%; *p* = 0.003) was significantly lower in the subcutaneous nitroglycerin group. However, no difference was found in hematoma (2.1% vs. 12.8%; *p* = 0.111). (Table 2).

**Table 2:** Results of radial artery cannulation in control group and nitroglycerin group

Variables	Control (*N* = 47)	Nitroglycerin (*N* = 47)	*p*-value
First attempt success rate of radial artery cannulation (%)	36/47 (76.6)	46/47 (97.9)	0.004
Procedure time to success within the first attempt (s)	171 ± 70	111 ± 18	< 0.001
Second attempt success rate within 10 min (%)	43/47 (91.5)	47/47 (100)	0.117
Overall number of attempts (1/2/3 attempts, *n*)	36/7/4	46/1/0	0.008
*Overall complication at first chosen radial artery*			
Vasospasm	15/47 (31.9%)	3/47 (6.4%)	0.003
Hematoma	6/47 (12.8%)	1/47 (2.1%)	0.111

Values are mean ± SD or number (proportion)

### Ultrasonographic measurements

**Fig. 3:**
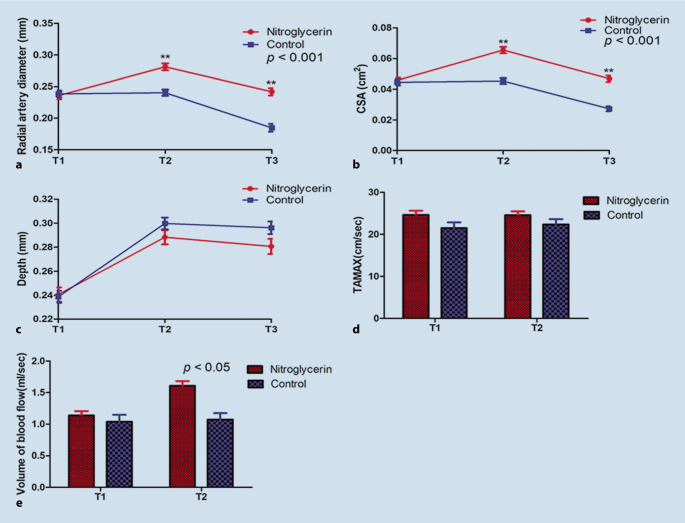
Results of ultrasonographic measurements: **a** radial artery diameter (mm), **b** cross-sectional area (CSA, cm^2^), **c** depth (mm), **d** time average maximal velocity (TAMAX, cm/sec), **e** volume of blood flow (ml/sec). T1 = baseline; T2 = 3 min after subcutaneous injection; T3 = immediately after radial artery cannulation

Over time, the diameter and CSA of radial artery showed significant differences between the two groups (*p* < 0.001). Compared with control group, the diameter and CSA of radial artery increased significantly at the T2 and T3 points in nitroglycerin group (*p* < 0.001), as well as the percentage change in radial artery diameter and CSA. No differences were found in the depth at the T1, T2 and T3 points between the groups (*P* = 0.289) (Table 3, Fig. 3a for diameter, Fig. 3b for CSA and Fig. 3c for depth).

**Table 3:** Results of ultrasonographic measurements in control group and nitroglycerin group

	Control (*N* = 47)	Nitroglycerin (*N* = 47)	*p*-value
Radial artery diameter (cm)			< 0.001
T1	0.24 ± 0.04	0.24 ± 0.04	
T2	0.24 ± 0.03	0.28 ± 0.04	
T3	0.18 ± 0.04	0.24 ± 0.04	
Percentage change of radial artery diameter at T2 (%)	0 (0–0)	20 (14–25)	< 0.001
Percentage change of radial artery diameter at T3 (%)	−30 (−20–−13)	0 (0–9)	< 0.001
CSA (cm^2^)			< 0.001
T1	0.045 ± 0.014	0.046 ± 0.012	
T2	0.045 ± 0.013	0.066 ± 0.014	
T3	0.027 ± 0.011	0.047 ± 0.014	
Percentage change of CSA at T2 (%)	0 (0–0)	43(33–60)	< 0.001
Percentage change of CSA at T3 (%)	−33 (−50–−25)	0 (0–20)	< 0.001
Depth (cm)			0.289
T1	0.24 ± 0.03	0.24 ± 0.04	
T2	0.30 ± 0.03	0.29 ± 0.04	
T3	0.30 ± 0.04	0.28 ± 0.04	
TAMAX (cm/s)			
T1	22 ± 9	25 ± 7	0.062
T2	22 ± 9	25 ± 6	0.157
Volume of blood flow(ml/sec)			
T1	1.04 ± 0.76	1.14 ± 0.47	0.447
T2	1.07 ± 0.69	1.61 ± 0.52	< 0.001

Values are mean ± SD, median (interquartile range) [range]. T1 = baseline; T2 = 3 min after subcutaneous nitroglycerin injection or saline injection; T3 = immediately after radial artery cannulation. *CSA* cross-sectional area, *TAMAX* time average maximal velocity

No differences were found in the TAMAX (*P* > 0.05) at the T1 and T2 points between the two groups. The volume of blood flow (*P* < 0.001) was significantly increased in the nitroglycerin group at the T2 point, but no difference was found at the T1 point (*P* = 0.447) (Table 3, Fig. 3d for TAMAX and Fig. 3e for blood flow).

## Discussion

The main finding of this study was that subcutaneous nitroglycerin increased the first attempt success rate of radial artery cannulation in women with gestational hypertension undergoing cesarean section. The relatively large diameter of the radial artery in the subcutaneous nitroglycerin group and improvement in palpability of radial pulse may contribute to the improved success rate, which is consistent with previous studies [15, 27]. We also found that subcutaneous nitroglycerin decreased cannulation times and overall number of cannulation attempts.

Successful radial artery cannulation was challenging for pregnant women with gestational hypertension. First, the anesthesiologists have less experience in ultrasound-guided arterial cannulation in this obstetrics and gynecology hospital. Second, the main challenge for successful radial artery catheterization in patients was the small diameter of the artery [15]. Generalized vasoconstriction is a feature of gestational hypertension [28]. Vasospasm or hematoma caused by failed attempts further reduces the artery’s inner diameter and the cannulation success rate [21, 23] As the radial artery was dominated by α1-adrenergic receptors [29], the radial artery was prone to vasospasm during cannulation attempts. Second, the effect of lidocaine injection on diameter of radial artery was paradoxical, one study showed expansion effects [27] and another study reported vasoconstriction [30]. On the other hand, radial artery was more sensitive to nitroglycerin as compared to other muscular arteries [31], and subcutaneously infiltrated nitroglycerin could result in marked radial artery vasodilation. This lead to avoidance of precannulation spasm and enhance palpability of the radial pulse, and thus improved the puncture of radial artery [27].

Up to 57% of cases would experience temporary vasospasm immediately after puncture of radial artery [32], and sustained vasospasm was reported in 4–20% during adult transradial cardiac catheterization [21]. A total of 15 patients (31.9%) experienced vascular spasm in the control group but only 3 (6.4%) patients in the nitroglycerin group. As a result, after the occurrence of vasospasm, the radial artery was successfully cannulated in 11 cases and failed in 4 cases of the 15 patients in the control group. The control group had a higher risk of vasospasm or complete occlusion. The radial artery catheterization was successful in 3 cases in the subcutaneous nitroglycerin group. In the control group, cannulation after vascular spasm failed in 4 (8.5%) patients. The relatively large radial artery inner diameter of subcutaneous nitroglycerin administration may increase the success rate of second attempts and reduce catheter failures. We found that subcutaneous nitroglycerin increases the diameter and CSA of radial artery, which is consistent with previous study [15]; however, few studies were concerned with the effect of subcutaneous nitroglycerin on TAMAX, or blood flow velocity. Additionally, a previous study has noticed that the depth of radial artery would affect ultrasound-guided cannulation [33]. A radial artery depth between 2 and 4 mm would get a higher first attempt success rate than those that are less than 2 mm or 4 mm or more in small pediatric patients [33]. Therefore, a small volume (0.5 ml) of local anesthetic or combined nitroglycerin solution was adopted to optimize the depth of radial artery. After subcutaneous injection, the mean radial artery depth of the two groups was in the range of 2–4 mm, and no significant difference was found between the two groups.

Different approaches, such as local [13], subcutaneous [34] and intra-arterial [26] approaches, were used in adult patients with radial artery dilatation administered with nitroglycerin. Intra-arterial nitroglycerin could decrease vasospasm and radial artery occlusion in adult cardiac patients, but not in arterial puncture patients, causing hematoma and reducing arterial diameter. Noninvasive vasodilation of the radial artery was the advantage of topical nitroglycerin cream; however, it should be smeared to the skin for at least 30 min before puncture of radial artery. Subcutaneous nitroglycerin stayed extravascular and acted locally for a longer duration to produce vasodilation than sublingual route of nitroglycerin administration [35]. Therefore, the authors chose subcutaneous nitroglycerin injection above the radial artery, which was the most effective method with minimal possible systemic side effects.

According to previous studies [27], 400–500 μg nitroglycerin is subcutaneously injected to facilitate radial artery cannulation without systemic hypotension in adult cardiac patients. Therefore, we determined the dose of 300 µg/0.3 ml nitroglycerin based on the pretest in the current study. In the current study, diameter of radial artery was significantly increased and no systemic side effect was observed in the subcutaneous nitroglycerin group, which is consistent with previous studies [15, 27, 36].

## Limitations

The current study has some limitations. First, the total sample was relatively small. Second, subcutaneous nitroglycerin should be injected near the target site as the radial artery dilatation occurs locally near the injection site. Third, subcutaneous injection may mask the ultrasound image of the radial artery, so it should be carefully injected under direct ultrasound without bubbles. Fourth, we evaluated radial artery diameter and distal perfusion only intraoperatively. As the timing of catheter removal varies with the type of surgery and clinical situation, we were unable to assess the radial artery diameter and distal perfusion after catheter removal.

## Conclusion

Subcutaneous nitroglycerin combined with local anesthetics before cannulation increased the first attempt success rate of radial artery cannulation and decreased the overall number of cannulation attempts in women with gestational hypertension and risks of intra-operative bleeding undergoing cesarean section. It also decreased cannulation times and overall number of vasospasms. It may be useful for those anesthesiologists with less experience in radial artery cannulation.

### Supplementary Information

Supplemental figure

## Abbreviations

ASA: American Society of Anesthesiologists
BMI: Body mass index
BP: Blood pressure
CSA: Cross-sectional area
TAMAX: Time-average maximum velocity

## Declarations

This study was approved by The Ethics Committee of Hangzhou Women’s Hospital (IRB:2021-K(9)-02) and written informed consent was obtained from all subjects participating in the study. All methods were performed in accordance with the relevant guidelines and regulations. Clinical trial number and registry URL: ChiCTR2100052981;http://www.chictr.org.cn, Principal investigator: Xin Men, date of registration: 7 November2021.

The supplement containing this article is not sponsored by industry.
