# Supplementary figures and images for: Subcutaneous nitroglycerin increased the success rate of radial artery cannulation in women with gestational hypertension undergoing cesarean section: A randomized controlled trial
Source: Anaesthesiologie. 2023 Mar 8;72(Suppl 1):28–35. doi: 10.1007/s00101-023-01264-6 (PMC10692033; doi:10.1007/s00101-023-01264-6)

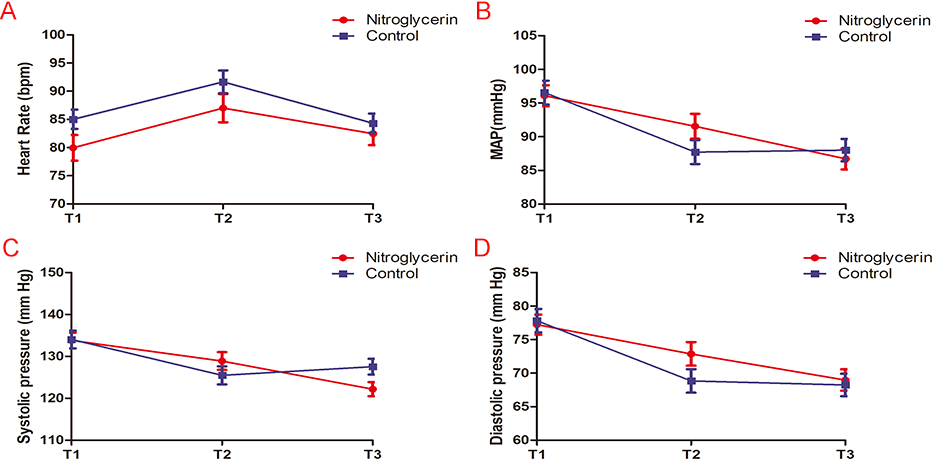

Supplement: Supplementary file 1 — Supplemental figure [file 101_2023_1264_MOESM1_ESM.tif]
